# Circumferential False Lumen Exclusion: A Technique for Indirect Cannulation of a Dissected Innominate Artery in Acute Type A Aortic Syndromes

**DOI:** 10.1055/a-2915-3215

**Published:** 2026-08-11

**Authors:** Merih Yilmaz, Ahmet Baltalarli, Abdullah Gokhun Alpua

**Affiliations:** 1 Department of Cardiovascular SurgeryPrivate Mugla Yucelen HospitalMuglaTurkiye; 2 Department of Cardiovascular SurgeryPrivate Denizli Cerrahi HospitalDenizliTurkiye

**Keywords:** acute Type A aortic syndrome, acute Type A aortic dissection, innominate artery dissection, innominate artery cannulation, antegrade cerebral perfusion, false lumen exclusion

## Abstract

Indirect innominate artery (IA) cannulation provides important advantages of central arterial inflow and antegrade cerebral perfusion in proximal aortic surgery but is often avoided when the artery is dissected. We describe a stepwise technique enabling indirect cannulation of a dissected IA through circumferential false lumen exclusion and adventitia–true lumen reapproximation. In nine patients with dissected IA undergoing this approach, cannulation was successfully achieved without in-hospital mortality or permanent neurological deficit.

## Introduction

Acute aortic syndromes (AAS) comprise acute aortic dissection, intramural hematoma, and penetrating aortic ulcer. When these entities involve the ascending aorta (Type A), they represent life-threatening conditions requiring emergent surgical intervention because of the risk of hemodynamic collapse, rupture, and irreversible end-organ injury.

The choice of arterial cannulation strategy in Type A AAS is primarily determined by the patient's hemodynamic status, supra-aortic branch involvement, and cerebral perfusion. Owing to the heterogeneous presentation of the disease, multiple arterial inflow strategies have been described, and no universally applicable approach has been established.

Current European Association for Cardio-Thoracic Surgery/Society of Thoracic Surgeons guidelines emphasize the importance of secure true lumen perfusion and early recognition of static malperfusion in acute Type A aortic dissection (ATAAD), noting that supra-aortic branch extension may significantly influence cannulation strategy. 1

ATAAD presents the greatest technical challenge for arterial inflow selection, as supra-aortic branch involvement is common.

Branch vessel involvement in ATAAD may manifest as dynamic compression or static obstruction, further complicating arterial inflow selection and cerebral protection strategy. 2

The principal objective of any cannulation strategy is the rapid and safe establishment of true lumen perfusion to reduce false lumen pressure before aortic rupture or irreversible end-organ damage occurs. Ideally, the selected inflow site should provide reliable true lumen access through the primary incision, minimize the risk of nerve injury or limb ischemia, and permit seamless transition to antegrade cerebral perfusion (ACP) without compromising right radial artery pressure monitoring. In addition, it should not require confirmation with transesophageal echocardiography or demand a high degree of technical expertise.

Although indirect cannulation of the innominate artery (IA) fulfills these criteria, it has traditionally been avoided when the artery itself is dissected because of concerns regarding technical difficulty and safety. 3 4 5

When the dissection plane is remote from the intended arteriotomy site, prevention of false lumen flow and graft anastomosis under clamping are relatively straightforward. In contrast, circumferential dissection poses a significant technical challenge, as pressurized false lumen flow displaces the true lumen away from the arteriotomy, compromising exposure and hemostasis, thereby making anastomosis cumbersome.

Accordingly, only a limited number of reports have described routine or systematic use of IA cannulation in ATAAD in the presence of supra-aortic branch vessel involvement. However, these reports either exclude cases with clear dissection of the IA itself or provide limited technical detail regarding control of false lumen pressurization and reapproximation of the compressed true lumen prior to arteriotomy. 4 5 6

Here we present a stepwise operative approach that enables controlled indirect cannulation of a dissected IA by achieving circumferential false lumen exclusion and adventitia–true lumen reapproximation prior to arteriotomy. For descriptive purposes, this approach is referred to as the Bilge technique.

## Operative Technique

**Video 1** Operative video demonstrating the Bilge technique in a patient with posterior and inferior wall dissection of the innominate artery (IA).


**Fig. 1:**
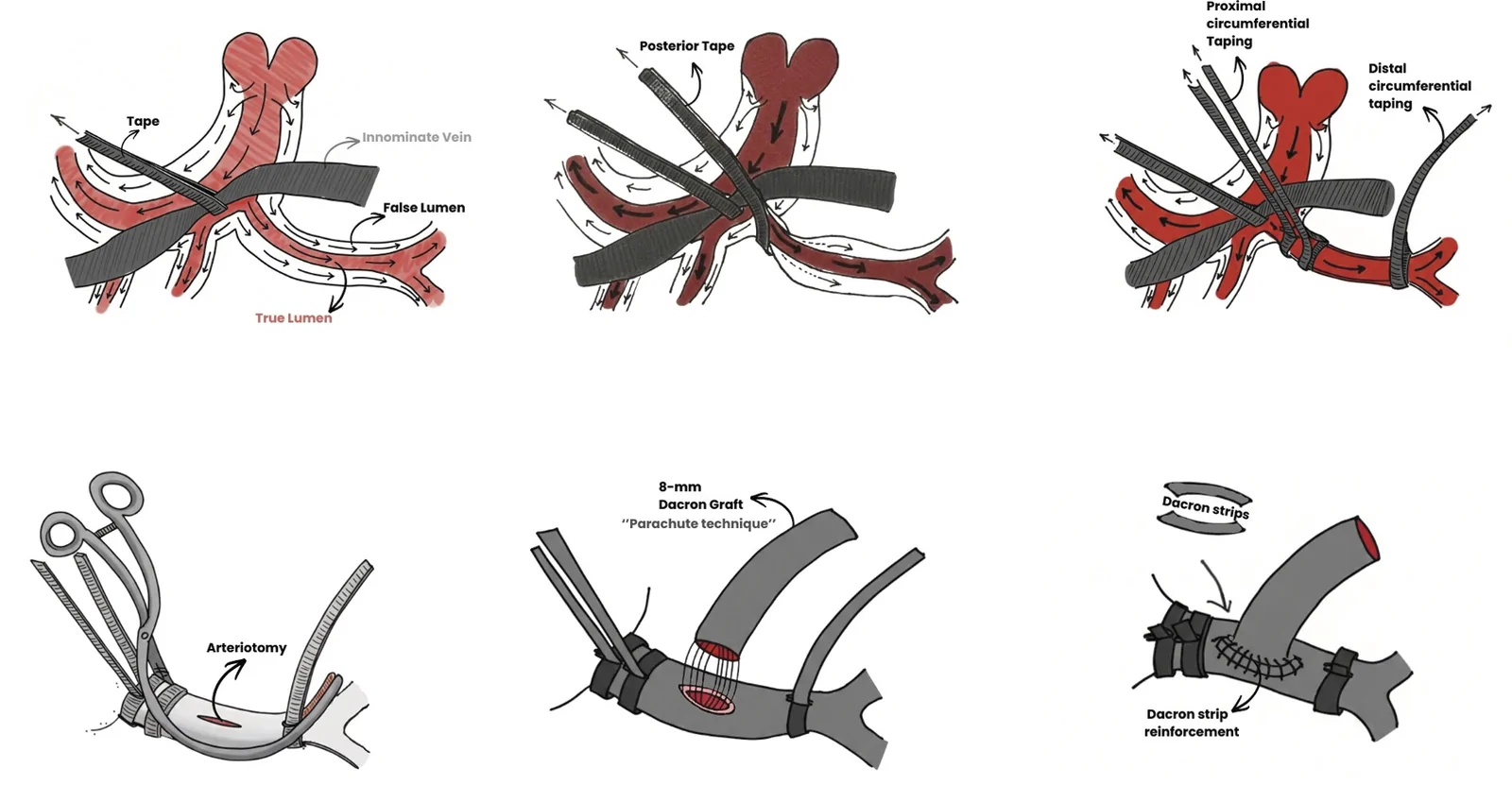
Central illustration demonstrating the stepwise application of the Bilge technique for indirect cannulation of a dissected IA, including surgical exposure, posterior decompression, circumferential taping to achieve false lumen exclusion, controlled clamping, and reinforced end-to-side anastomosis of an 8-mm Dacron side graft.

The operative steps are schematically illustrated in Fig. 1 , with key intraoperative steps demonstrated in Video 1 (available in the online version only), recorded in a patient with posterior and inferior wall dissection of the IA.

### Step 1. Surgical Exposure

Following median sternotomy, the pericardium is deliberately left unopened. The innominate vein is mobilized and encircled with a broad tape. Gentle traction toward the left and inferior provides adequate exposure of the IA without vein transection. Overlying tissues are divided, and the IA is exposed using an army–navy retractor.

### Step 2. Posterior Decompression

A broad tape is passed posterior to the proximal IA using a right-angled clamp guided by the surgeon's index finger. Suspension of this tape reduces false lumen pressure at the arterial origin.

### Step 3. Circumferential False Lumen Exclusion

**Video 2** Circumferential proximal and distal taping of the IA demonstrating controlled arterial stabilization before anastomosis. IA, innominate artery.


A second broad tape is wrapped circumferentially around the proximal IA twice and gently snared, occluding the false lumen and reapproximating the adventitia to the true lumen. The snare is secured with a hemoclip. A similar circumferential taping maneuver is then performed on the distal IA using a third broad tape, stabilizing the arterial wall and completing false lumen exclusion. Circumferential proximal and distal taping is demonstrated in Video 2 (available in the online version only).

### Step 4. Management of False Lumen Thrombosis (Selected Cases)

**Video 3** Controlled evacuation of false lumen thrombus during preparation of the dissected IA under side clamping. Preoperative computed tomography angiography (CTA) demonstrates obstruction of the right common carotid artery (RCCA). The video shows color change and intramural hematoma of the IA, followed by controlled adventitiotomy and thrombus evacuation. Bleeding is controlled by gentle clamp adjustment. Postoperative CTA confirms restoration of flow in the RCCA.


In selected cases with preoperative or intraoperative suspicion of false lumen thrombosis, an adventitiotomy is performed before final tightening of the snares. Residual false lumen pressure facilitates evacuation of thrombotic material, restoring true lumen patency ( Video 3 , available in the online version only).

### Step 5. Clamping and Arteriotomy

One or two vascular clamps are gently applied over the taped segments, occluding both true and false lumens and providing a controlled, bloodless field. A longitudinal anterior arteriotomy is performed at the site of adventitia–true lumen reapproximation.

### Step 6. Side-Graft Anastomosis

**Video 4** End-to-side anastomosis of an 8-mm Dacron graft to a circumferentially dissected IA following controlled arteriotomy. Preoperative computed tomography angiography (CTA) and postoperative CTA are shown to demonstrate restoration of branch vessel perfusion. After completion of the anastomosis, circumferential tapes are briefly relaxed to demonstrate arterial stability and anastomotic integrity.


An 8-mm Dacron graft is anastomosed end-to-side using a parachute technique. Two narrow Dacron strips are interposed between the suture line and adventitia to reinforce the anastomosis. The graft is cannulated, cardiopulmonary bypass is initiated, and systemic perfusion is followed by transition to ACP as required. Application of this approach in a circumferentially dissected IA is shown in Video 4 (available in the online version only).

## Clinical Experience

Between 2014 and 2026, 134 aortic operations were performed by two surgeons across three institutions. After 2016, the IA became the preferred arterial inflow site in our practice, and 109 patients underwent indirect IA cannulation during this period. During the same interval, 23 patients underwent surgery for acute Type A aortic syndromes.

IA cannulation was performed in 19 cases (83%). In the remaining four patients, alternative cannulation strategies were employed: femoral artery cannulation in one hemodynamically unstable re-redo patient; ascending aortic cannulation in one patient with aberrant right subclavian artery and ruptured type III dissection; and right axillary artery cannulation in two ATAAD cases prior to 2016.

Among the 19 IA cannulation cases, 9 patients demonstrated partial or circumferential dissection of the IA and underwent indirect cannulation using circumferential false lumen exclusion. Clinical and operative characteristics of these patients are summarized in Table 1 .

**Table 1:** Clinical and operative characteristics of patients with acute Type A aortic syndrome and dissected innominate artery undergoing cannulation using the Bilge technique

Patient	Age/sex	Aortic syndrome	IA dissection pattern	Branch involvement	CPB (min)	ACC (min)	Procedure	Permanent neurological deficit	Hospital mortality
1	58/M	Type 1 AD	Circumferential dissection and true lumen compression with false lumen thrombosis	Static obstruction (IA false lumen thrombus–RCCA, RSCA), dissection (LCCA, LSCA)	200	98	IA thrombus removal + Asc Ao replacement	No	No
2	63/M	Type 1 AD (limited intimal tear) with distal IMH	Posterior–inferior IMH	None	245	145	Asc Ao replacement	No	No
3	48/M	Type 1 AD (with chronic Type 3 component)	Posterior–inferior wall dissection	Dissection (RSCA, RCCA)	279	172	Fenestration + total arch replacement + AV resuspension	No	No
4	57/M	Type 1 AD (contained rupture)	Posterior–inferior wall dissection	Dissection (RSCA, RCCA)	208	109	Asc Ao replacement	No	No
5	35/M	Type 2 AD	Posterior wall dissection	None	303	206	Bentall + hemiarch replacement + IA translocation	No	No
6	45/M	Type 1 AD	Circumferential dissection	Dynamic compression (RCCA)	212	114	Asc Ao replacement + AV resuspension	No	No
7	48/M	Type 1 AD	Circumferential dissection	Dynamic compression (RSCA), dissection (RCCA, LCCA, LSCA)	280	190	Asc Ao replacement + AV resuspension	No	No
8	77/M	Type 2 AD (contained rupture)	Posterior wall dissection	None	240	150	Asc Ao replacement + AV resuspension + IA translocation	No	No
9	48/M	Type 1 AD	Posterior–inferior wall dissection	Dynamic compression (RSCA), dissection (RCCA, LSCA)	360	270	Bentall + hemiarch replacement + CABG ×1 (RCA)	No	No

Abbreviations: ACC, aortic cross-clamp; AD, aortic dissection; Asc Ao, ascending aorta; AV, aortic valve; CABG, coronary artery bypass grafting; CPB, cardiopulmonary bypass; IA, innominate artery; IMH, intramural hematoma; LCCA, left common carotid artery; LSCA, left subclavian artery; M, male; RCA, right coronary artery; RCCA, right common carotid artery; RSCA, right subclavian artery.

All patient records, operative reports, and available preoperative and postoperative imaging studies were retrospectively reviewed. Supra-aortic branch involvement was classified based on comparison of contrast-enhanced preoperative imaging, intraoperative findings, and postoperative imaging when available. Dynamic compression was defined as absence of preoperative branch opacification with restoration of flow postoperatively without surgical thrombectomy. Static obstruction was defined as flow compromise attributable to false lumen thrombosis requiring intraoperative evacuation. Branch dissection without flow compromise was recorded when contrast opacification was preserved despite arterial wall dissection.

In one patient, static obstruction at the IA bifurcation due to false lumen thrombosis was identified intraoperatively. Following evacuation of thrombotic material ( Video 3 , available in the online version only), improvement in right radial arterial waveform was observed. Postoperative contrast-enhanced imaging confirmed resolution of static obstruction in the right subclavian artery and right common carotid artery.

Procedures performed included ascending aortic replacement, hemiarch replacement, total arch replacement, and composite root procedures as indicated by individual pathology. Cardiopulmonary bypass and aortic cross-clamp times are detailed in Table 1 .

Overall, in-hospital mortality for the entire acute Type A cohort was 2/23 (8.7%). One death occurred in a patient with acute Type A dissection and distal intimointimal intussusception who required total arch replacement and Bentall procedure. The IA was not dissected in this case. This patient is not included in Table 1 , as the table is limited to patients with dissected IA. The patient experienced postoperative cardiac arrest shortly after surgery and subsequently developed global brain injury. The second death occurred in a hemodynamically unstable re-redo patient who required femoral artery cannulation under ongoing cardiopulmonary resuscitation.

Importantly, there was no in-hospital mortality and no permanent neurological deficit among patients with dissected IA undergoing indirect cannulation.

## Discussion

Dissection of the IA has traditionally been regarded as a contraindication to cannulation because of concerns related to uncontrolled false lumen pressurization, difficulty in achieving a bloodless field, and the potential risk of arterial rupture. 3 4 Without prior reduction of false lumen pressure and adventitia–true lumen reapproximation, pressurized false lumen flow may displace the true lumen away from the arteriotomy site, rendering graft anastomosis technically cumbersome.

The Bilge technique may address these concerns by achieving circumferential false lumen exclusion and adventitia–true lumen reapproximation prior to arteriotomy. By reducing false lumen pressure proximally and distally, a stable and controlled operative field can be established, facilitating a controlled side-graft anastomosis. Circumferential false lumen exclusion allows indirect cannulation of a dissected IA while preserving the advantages of central arterial inflow.

Among the nine patients with dissected IA undergoing indirect cannulation using this approach, no in-hospital mortality or permanent neurological deficit was observed. No case required conversion to an alternative arterial inflow site.

Rather than representing a bailout maneuver, circumferential false lumen exclusion allows controlled extension of established IA cannulation principles to selected cases with dissected arterial anatomy. In our experience, suitability for controlled false lumen exclusion is determined by preoperative imaging and intraoperative assessment of the dissected arterial wall, particularly the absence of prohibitive calcific plaque and the feasibility of achieving stable proximal and distal arterial control.

An additional benefit is the ability to manage false lumen thrombosis before cardiopulmonary bypass.

Given the descriptive and retrospective nature of this report and the limited number of dissected IA cases, these observations should be interpreted within this technical context.

Controlled circumferential false lumen exclusion enables technically feasible and reproducible indirect cannulation of a dissected IA in acute Type A aortic syndromes. The Bilge technique may expand the applicability of IA cannulation in selected patients with dissected supra-aortic vessels while preserving the advantages of central arterial inflow.
